# Thermal Field and High-Temperature Performance of Epoxy Resin System Steel Bridge Deck Pavement

**DOI:** 10.3390/ma18133109

**Published:** 2025-07-01

**Authors:** Rui Mao, Xingyu Gu, Jiwang Jiang, Zhu Zhang, Kaiwen Lei

**Affiliations:** School of Transportation, Southeast University, Nanjing 211189, China; 220233494@seu.edu.cn (R.M.); jiang_jiwang@seu.edu.cn (J.J.);

**Keywords:** steel bridge deck pavement, Epoxy Resin System, thermal field, high-temperature mechanical properties

## Abstract

Epoxy Resin System (ERS) steel bridge pavement, which comprises a resin asphalt (RA) base layer and a modified asphalt wearing course, offers cost efficiency and rapid installation. However, the combined effects of traffic loads and environmental conditions pose significant challenges, requiring greater high-temperature stability than conventional pavements. The thermal sensitivity of resin materials and the use of conventional asphalt mixtures may weaken deformation resistance under elevated temperature conditions. This study investigates the thermal field distribution and high-temperature performance of ERS pavements under extreme conditions and explores temperature reduction strategies. A three-dimensional thermal field model developed using finite element analysis software analyzes interactions between the steel box girder and pavement layers. Based on simulation results, wheel tracking and dynamic creep tests confirm the superior performance of the RA05 mixture, with dynamic stability reaching 23,318 cycles/mm at 70 °C and a 2.1-fold improvement in rutting resistance in Stone Mastic Asphalt (SMA)-13 + RA05 composites. Model-driven optimization identifies that enhancing internal airflow within the steel box girder is possible without compromising its structural integrity. The cooling effect is particularly significant when the internal airflow aligns with ambient wind speeds (open-girder configuration). Surface peak temperatures can be reduced by up to 20 °C and high-temperature durations can be shortened by 3–7 h.

## 1. Introduction

Steel bridges, such as cable-stayed and suspension bridges, are widely used for long spans due to their high load-bearing capacity, fabrication precision, and fast installation. The steel bridge deck pavement acts both as a structural layer and a service surface, protecting the orthotropic steel deck from corrosion and mechanical damage while providing durable performance for traffic. Its thermomechanical behavior directly affects vehicular safety, ride comfort, and the long-term durability of the bridge. Key factors that influence its performance include the modulus compatibility between pavement materials and the steel deck, surface smoothness, and resistance to fatigue under environmental and traffic loads [1]. Unlike conventional subgrade-supported pavements, steel bridge deck pavements exhibit much more complex thermo-mechanical coupling behavior, characterized by multi-axial stress states under moving loads and surface temperatures rising to 60–70 °C during summer. This unique service environment results from the combined effects of constrained thermal expansion in orthotropic steel decks and amplified dynamic vehicle loading on low-stiffness pavement systems [2]. Conventional bridge deck systems typically use asphalt concrete pavements with bituminous or resin-based bonding materials, both of which show significant temperature-dependent performance variations [3]. Under different environmental factor and vehicle load conditions, steel bridge deck pavements are susceptible to distress, such as rutting and displacement, imposing strict requirements on the materials used for the pavement layer [4,5,6].

As a result, bridge deck pavement materials must meet enhanced thermal stability criteria. Three main conventional systems are commonly used, including cast asphalt mixtures, double-layer modified asphalt systems, and epoxy asphalt composites [7]. The Guss Asphalt and double-layer modified asphalt systems exhibit insufficient high-temperature performance in practical applications. In contrast, the epoxy asphalt pavement system, while offering superior performance, is limited by significantly higher implementation costs [8,9,10]. Researchers have also attempted to use poly methyl methacrylate and polyurethane asphalt materials in structural layers. While the poly methyl methacrylate bridge deck pavement system exhibits excellent performance, it incurs higher costs. Conversely, the polyurethane asphalt system offers better mechanical properties and easier construction, but its high-temperature performance is relatively inferior [11,12].

However, the high thermal conductivity of steel, its sensitivity to temperature changes [13], and the complex service environment of steel box girder bridges result in significant differences in the service conditions of the asphalt mixture in the pavement layer compared to traditional asphalt pavements. To accurately assess the temperature impact on steel bridge decks, it is necessary to determine an appropriate temperature field for the bridge deck pavement layer [14]. Although in situ monitoring provides a realistic characterization of bridge deck pavement temperature fields, its application is often constrained by insufficient data sampling and limited engineering feasibility. Specifically, temperature field detection on in-service bridge pavements faces significant operational challenges due to traffic interference and limited opportunities for sensor deployment [15]. Consequently, researchers have adopted computational modeling approaches to simulate temperature field distributions, thereby successfully deriving validated evolution patterns of pavement layer temperature gradients that align with empirical observations [16,17,18,19]. Liu et al. developed a thermomechanical coupling model for steel box girders grounded in transient heat transfer principles, implementing the element activation/deactivation technique to precisely replicate the phased construction process of Guss Asphalt mixture placement, with particular emphasis on thermal–structural interaction mechanisms during material deposition [20]. Zhou et al. formulated a three-dimensional coupled heat transfer model for steel box girder–pavement layer systems using ABAQUS finite element analysis, integrating thermal conduction principles to quantify interfacial thermomechanical interactions. Their computational framework successfully decoupled the thermal response mechanisms between structural components under diurnal temperature fluctuation conditions, revealing critical phase lag characteristics in heat flux propagation across material interfaces [21]. However, current studies on temperature field analysis have primarily focused on external environmental factors, with limited research on the internal thermal conditions within steel box girders.

The ERS bridge deck pavement system employs a three-layer structure that comprises an Epoxy-Bonding Chip Layer (EBCL), cold-mixed resin asphalt mixture (RA), and Stone Mastic Asphalt (SMA) [10]. The ERS pavement system demonstrates superior performance with ambient temperature construction feasibility, reduced technical demands, and rapid serviceability after minimal curing. Notably, its exceptional waterproofing characteristics, combined with its facile maintenance for distress remediation and cost-effectiveness, endow this system with substantial application potential in bridge engineering practice. Current practices in ERS bridge deck pavement applications predominantly rely on empirical experience and precedent construction cases for parameter determination across structural layers. The ERS bridge deck pavement system uses resin materials as the core, with the upper layer typically consisting of SMA mixtures. This system is characterized by susceptibility to high-temperature distress. However, there is limited research on this topic, so it is important to focus on the impact of temperature on the performance of the ERS bridge deck pavement system [22,23]. When investigating the performance of the ERS pavement system, it is essential not only to validate the high-temperature performance of the materials in each structural layer but also to study the overall high-temperature performance of the composite structure, ensuring that it aligns with the actual bridge deck environment.

Therefore, this paper uses finite element analysis software COMSOL Multiphysics 6.2 (trial version) to establish a three-dimensional model of the steel box girder and pavement layer. Using simulations, the high-temperature field of the bridge deck pavement layer at different times of the day is calculated, while also considering the influence of airflow velocity inside the steel box girder on the temperature field. Unlike most previous studies that considered only external environmental temperatures, this study incorporates internal airflow effects. In addition to verifying the high-temperature performance of pavement materials through rutting tests, this study further combines the simulated temperature field with modified dynamic creep tests conducted on the composite structure under extreme thermal conditions, aiming to evaluate the overall high-temperature deformation resistance of the ERS steel bridge deck pavement system. Prior studies often focused solely on the material-level performance of epoxy–asphalt composites, whereas this work emphasizes structural-level validation of the composite system under realistic thermal conditions. The aim is to verify the rationality of the ERS pavement system as a dual-layer heterogeneous structure capable of resisting high-temperature deformation on steel bridge decks. Furthermore, measures to mitigate high-temperature distress in the steel bridge deck pavement layer, without altering the stiffness and strength of the box girder, are proposed.

## 2. Materials and Methods

### 2.1. Materials

**Figure 1:**
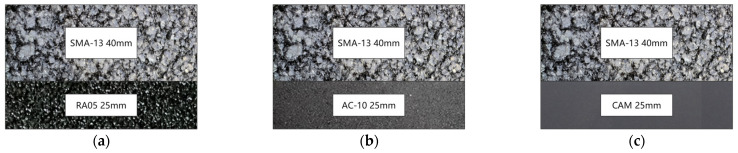
Schematic diagram of the structure. (**a**) Structure of SMA-13 and RA05; (**b**) Structure of SMA-13 and AC-10; (**c**) Structure of SMA-13 and CAM.

This study compares the high-temperature performance of three pavement structures including SMA-13 with RA05 protective layer, conventional SMA-13 with AC-10 asphalt concrete, and SMA-13 combined with crack-attenuating mixes (CAM). The investigation evaluates the effects of three distinct base course configurations on composite structural performance. The schematic diagrams of the three composite structures are shown in Figure 1.

#### 2.1.1. Mixture Design of Wearing Course

In the ERS pavement system, the wearing course consists of SMA-13 mixture. The asphalt mixture was fabricated using Styrene–Butadiene–Styrene (SBS)-modified asphalt binder. Based on relevant standards, the corresponding technical indices are shown in Table 1.

**Figure 2:**
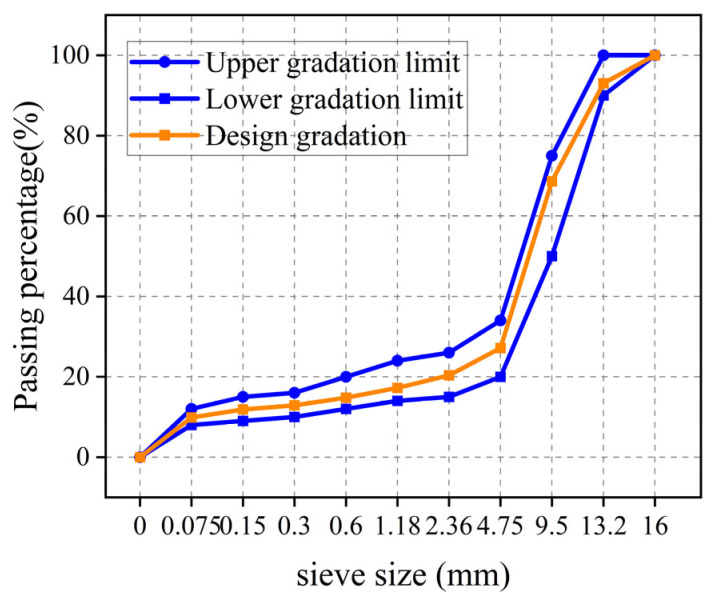
Gradation characteristics of SMA-13.

The corresponding gradation curve of SMA-13 is graphically presented in Figure 2.

Standard Marshall specimens were fabricated, and the relevant technical parameters of the asphalt mixture were evaluated. Table 2 summarizes the technical parameters of SMA-13 asphalt mixture under the optimum asphalt–aggregate ratio.

#### 2.1.2. Mixture Design of Base Course

In the ERS bridge deck pavement system, the RA05 mixture serves as the primary base course material, allowing for ambient temperature mixing, paving, and compaction with rapid strength development. The cured material demonstrates high strength, high modulus, low void content, rutting resistance, and corrosion resistance, functioning as the main load-bearing structure in ERS pavement systems.

The RA binder (provided by Ningbo Tianyi Steel Deck Pavement Technology Co., Ltd., Ningbo, China) consists of two components. Component A (main agent) is a mixture of epoxy resin and petroleum asphalt, while Component B (curing agent) contains modified polyamine curing agent and petroleum asphalt. During preparation, Components A and B are first individually stirred using electric mixers at 1200 rpm for approximately 60 s and then mixed at a 1:1 ratio with continuous stirring for 180 s to ensure homogeneity. The components undergo cross-linking curing at ambient temperature, forming a solid gel-like material with adequate strength and asphalt-comparable viscosity, enabling effective combination with aggregates to create resin–asphalt mixtures. The technical parameters of the RA binder are presented in Table 3.

The RA05 mixture was formulated with 8.0% binder–aggregate ratio, and the technical parameters of RA05 mixtures are summarized in Table 4.

The remaining two pavement structures employ AC-10 and CAM materials for their base course configurations, respectively. AC-10 demonstrates superior deformation resistance and durability characteristics [26]. The CAM material, developed in Texas, USA, effectively controls both rutting and reflection cracks while maintaining pavement flexibility [27].

The technical parameters of AC-10 asphalt concrete and CAM mixtures are summarized in Table 5.

**Figure 3:**
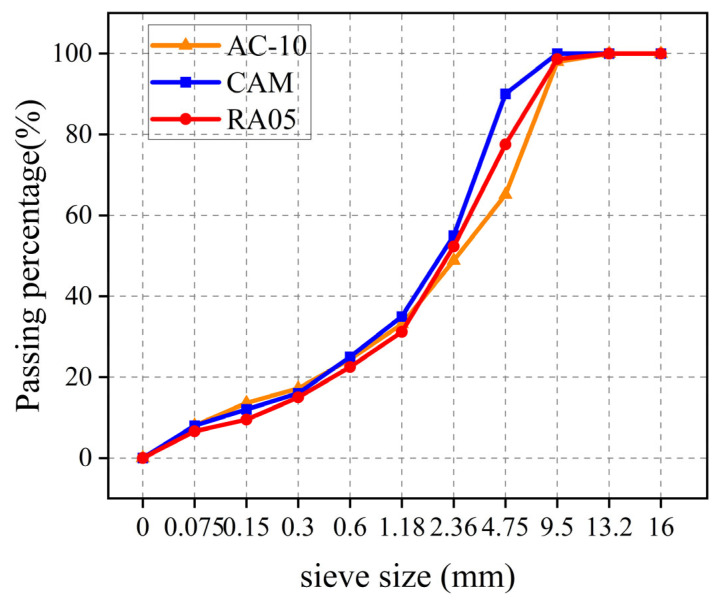
Gradation characteristics base course material.

The gradation curve of RA05, AC-10 and CAM are graphically presented in Figure 3.

### 2.2. Methods

#### 2.2.1. High-Temperature Performance Tests

**Figure 4:**
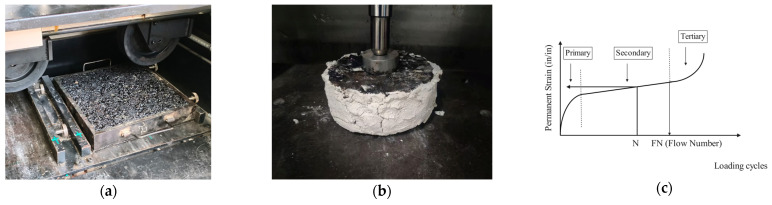
High-temperature performance tests. (**a**) Rutting test; (**b**) High-temperature dynamic creep test; (**c**) Typical relationship between total cumulative plastic strain and loading cycles.

The rutting test, as illustrated in Figure 4a, serves as the current standard methodology for high-temperature stability evaluation in pavement engineering. Dynamic stability (DS) is adopted in this study as the evaluation criterion, with higher values indicating improved rutting resistance and enhanced high-temperature performance of the materials.

Following comprehensive high-temperature performance evaluations of constituent materials, subsequent analysis focuses on composite system characterization. The high-temperature dynamic creep test, based on the viscoelastic properties of asphalt mixtures, enables the quantitative evaluation of material performance under varying temperature and stress conditions. Furthermore, this methodology accurately simulates dynamic loading variations, closely replicating real-world service conditions involving simultaneous thermal and mechanical stresses. Consequently, the high-temperature dynamic creep test is recommended as the principal experimental protocol for assessing composite structural performance under varying thermomechanical coupling effect conditions. Experimental measurements of axial strain were systematically recorded to establish the relationship between cumulative permanent deformation and loading cycles [28,29,30,31]. This strain–cycle curve effectively captures the progressive deterioration pattern of the material under repeated load applications.

To better investigate the high-temperature performance of the bridge deck pavement composite structure, this study will refine the experiment based on the actual bridge deck pavement structure, as shown in Figure 4b.

The experiment involves measuring axial strain to determine the relationship between cumulative permanent strain and the number of load cycles. As shown in Figure 4c, this relationship consists of three stages: in the initial stage, permanent strain accumulates rapidly, but the accumulation rate gradually decreases; in the second stage, the accumulation rate remains constant; and in the third stage, the accumulation rate of permanent strain increases sharply again. To focus on variations in the creep characteristics of the structure, $N_{ps}$ represents the number of load cycles applied at the start of the second stage. The flow number (FN) is used in this study as the evaluation metric for the composite structure’s high-temperature performance, where higher values correlate with superior rutting resistance [28].

#### 2.2.2. Modified Dynamic Creep Test

To investigate the high-temperature performance of the bridge deck pavement composite structure, the improved experiment will replicate the actual bridge deck pavement structure. The wearing course and base course will be combined to prepare the specimens. The specimen prepared by using rotational compaction method can effectively simulate the actual construction process of steel bridge deck pavement, including the compaction in construction, the thickness control of the overall structure, and the setting order of the structural layer. It can also enhance compaction uniformity in the structural layers and prevent issues such as cracking, delamination, and void formation [32]. The process begins with the preparation of RA05 mixture specimens (Φ100 × 25 mm) as the base course. Next, a 1 kg/m^2^ layer of SBS-modified asphalt will be applied as the bonding layer on the surface. The SMA-13 mixture will then be prepared and placed on top of the base course, which is formed using a rotational compactor to achieve a wearing course height of 40 mm. After curing, a composite specimen (Φ100 × 65 mm) will be obtained, differing from the standard dynamic creep specimen (Φ100 × 150 mm).

To simulate the thermal gradient characteristics of steel bridge decks, considering both surface heat conduction and structural insulation effects, thermal insulation coatings were applied to the lateral and bottom surfaces, allowing heat transfer only through the SMA-13 upper layer. The specimens were subjected to 4 h of thermal equilibrium with the SMA-13 surfaces facing upward before testing.

### 2.3. Modeling and Parameterization

#### 2.3.1. Thermal Field Analytical Methodology

**Figure 5:**
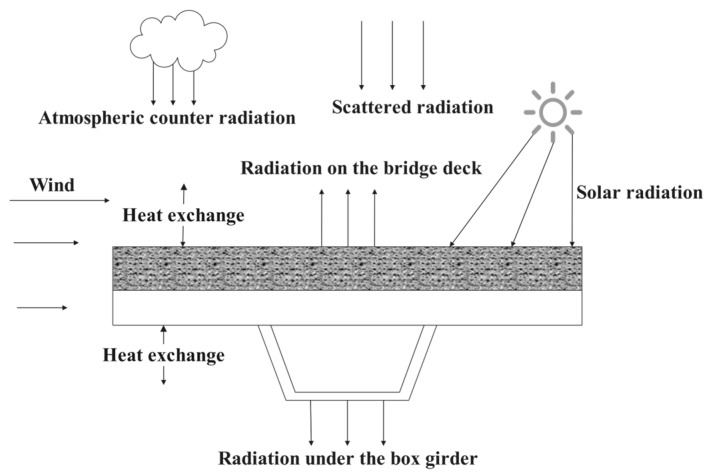
Environmental effects on thermal fields of steel box girder.

In addition to vehicular loading, steel bridge deck pavement systems are simultaneously subjected to coupled environmental actions, including solar radiation, ambient temperature fluctuations, and precipitation impacts. Figure 5 schematically illustrates the multi-physics interaction mechanisms between these environmental factors and the pavement thermal field [33].

Three primary thermal factors significantly influence the system, involving effective radiation between steel box girders and pavement layers, solar radiation exposure, and convective heat exchange with the air. This study analyzes temperature field variations in steel bridge deck pavements under these combined effects using thermal field computational theory.

The thermal boundary conditions for pavement layers and steel bridge decks under transient thermal field theory can be defined as follows [34,35]:

(1) $$-K{\left.\frac{\partial T}{\partial y}\right|}_{y=0}=h_{c}f\left[{\left.T_{1}\left(t\right)\right|}_{y=0}-T_{a}\right]+q\left(t\right)+\varepsilon \sigma \left[{\left(T_{a}-T_{Z}\right)}^{4}-{\left({\left.T_{1}\left(t\right)\right|}_{y=0}-T_{Z}\right)}^{4}\right],$$

where K = thermal conductivity (J∙m^−1^∙h^−1^∙°C^−1^);

h_c_ = the convective heat transfer coefficient (W∙m^−2^∙°C^−1^);

T_1_(t) = temperature distribution within structural components;

q(t) = solar radiation intensity;

ε = the surface emissivity coefficient;

σ = the Stefan–Boltzmann constant (σ = 2.041092 × 10^−4^);

T_Z = absolute zero temperature;

T_a_ = ambient air temperature.

For steel box girder sections without solar exposure (bottom/interior surfaces), the thermal boundary conditions are expressed as follows:

(2) $$-K{\left.\frac{\partial T}{\partial y}\right|}_{y=h}=h_{c}f\left[{\left.T_{1}\left(t\right)\right|}_{y=h}-T_{a}\right]+\varepsilon \sigma \left[{\left(T_{a}-T_{Z}\right)}^{4}-{\left({\left.T_{1}\left(t\right)\right|}_{y=h}-T_{Z}\right)}^{4}\right],$$

(3) $$-K{\left.\frac{\partial T}{\partial y}\right|}_{y=y_{0}}=\varepsilon \sigma \left[{\left(T_{a}-T_{Z}\right)}^{4}-{\left({\left.T_{1}\left(t\right)\right|}_{y=y_{0}}-T_{Z}\right)}^{4}\right],$$

#### 2.3.2. Modeling Framework and Parameters

This study adopts the Yangmeizhou Bridge in Xiangtan City, Hunan Province, China as the engineering prototype. Situated in a subtropical monsoon humid climate zone (16.7–17.4 °C annual average temperature), the bridge experiences prolonged summer heat with mean wind speeds of 2.4 m/s (maximum 20 m/s).

The 1108 m-long cable-stayed bridge features a 658 m steel box girder main span connected by transverse steel beams, constituting the longest single-span crossing over the Xiangjiang River. The bridge deck configuration comprises six-lane bidirectional traffic lanes with dedicated non-motorized and pedestrian pathways. The pavement system employs an Epoxy Resin System (ERS)-layered structure consisting of SMA-13 wearing course, RA05 base course, and Epoxy-Bonded Chip Layer (EBCL) adhesion stratum.

**Figure 6:**
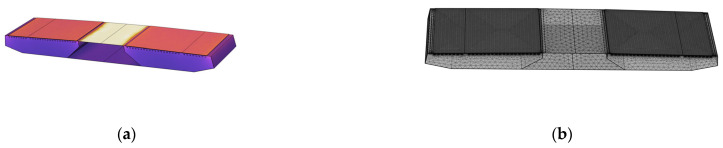
Configuration of bridge segment model. (**a**) Thermal field simulation diagram; (**b**) Grid division diagram.

A finite element model of the main span structure was developed using finite element analysis software, incorporating these geometric and material parameters. As shown in Figure 6b, when dividing the grid, the pavement layer is divided in a refined way, and the other parts are divided into regular sizes.

Based on previous research, the key technical parameters are shown in Table 6 [36,37,38].

The thermal field was analyzed using the developed bridge segment model. Radiation and convective boundary conditions were applied to the pavement layer surfaces and steel box girder exteriors, with other surfaces maintained as adiabatic boundaries. The simulation assumed (1) homogeneous and isotropic pavement layer materials; (2) uniform temperature distribution along the bridge axis; and (3) continuous heat transfer between structural layers. Additionally, it is assumed that the interlayer contact between pavement layers and the contact between the pavement layer and the steel bridge deck are perfectly continuous, with the geometric dimensions of the bonding layer neglected.

## 3. Results and Discussion

### 3.1. Thermal Field Model

**Figure 7:**
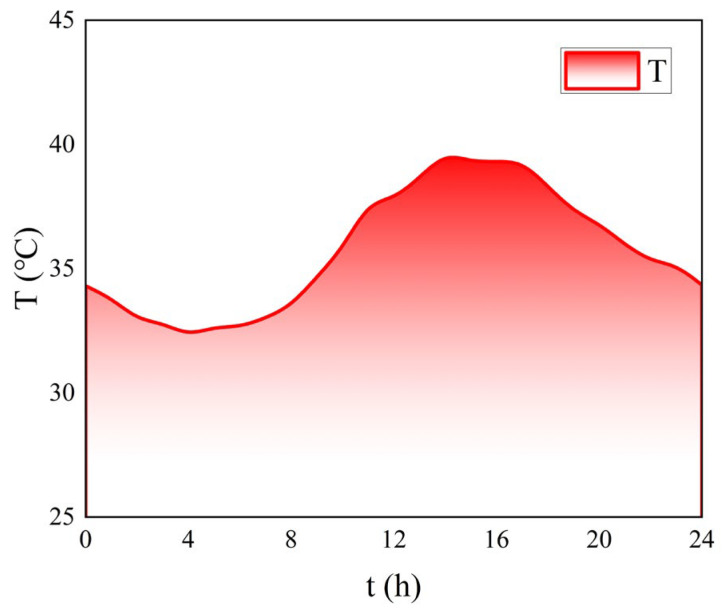
Temperature variation pattern.

The rutting resistance of pavement layers under extreme heat conditions serves as a critical performance indicator. This investigation specifically examines the thermal behavior of pavement structures during summer peak temperatures in Xiangtan City, Hunan Province, China. According to the previous research and local weather station data, the temperature data in summer are obtained. Representative temperature data from 00:00 to 24:00 on a typical July day (Figure 7) were adopted as simulation parameters, with recorded extremes of 40 °C (maximum), 32 °C (minimum), and 36 °C (average).

**Figure 8:**
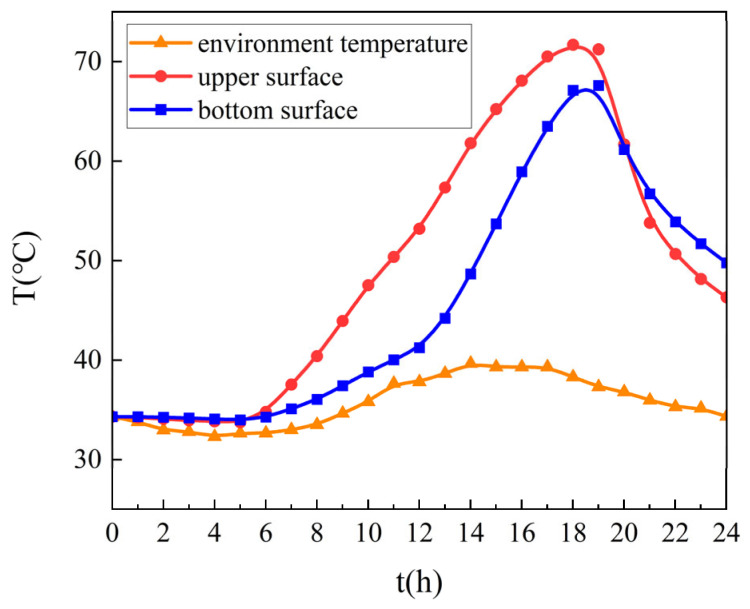
Thermal field simulation results.

The internal airflow velocity in steel box girders was fixed at 0 m/s. Temperature distributions at both the upper surface of pavement layers and the lower surface of pavement layers (the interface between the pavement layer and the steel box girder) were calculated, with corresponding results presented in Figure 8.

Analysis of Figure 8 indicates that pavement layers maintain higher temperatures than ambient air, with the upper surface reaching peak values of 70 °C due to solar radiation and convective heating. Sustained thermal exposure exceeding 50 °C for nearly 10 h was observed on the surface, while the bottom layer reached 67 °C, thereby critically challenging the material durability.

The thermal field simulation results demonstrated a temporal delay in temperature peaks (approximately 17:00), showing 1.5 h phase lag between upper surface and bottom surface layers. Notably, thermal inversion occurred after 19:00 as base temperatures surpassed surface readings, attributed to restricted convective heat transfer within sealed interlayers creating insulation effects.

### 3.2. High-Temperature Performance Evaluation of Pavement Layers

Comparative analysis was conducted on three pavement systems, including SMA-13+RA05, SMA-13+AC-10, and SMA-13+CAM. The evaluation specifically focused on their thermal stability under elevated temperature conditions through controlled laboratory testing. Thermal field analysis reveals significantly elevated temperature conditions in steel bridge deck pavement layers compared to conventional scenarios, with peak temperatures reaching 70 °C at the upper surface and 65 °C at the bottom surface. This thermal profile justification establishes 70 °C as the laboratory testing temperature for performance evaluation [24].

#### 3.2.1. Comparison of Different Mixtures

Initial evaluations were conducted on SMA-13 and RA05 mixtures to assess their high-temperature performance characteristics. Subsequent comparative analysis with benchmark materials (CAM and AC-10 mixtures) revealed significant performance differences. Three parallel test groups (*n* = 3) were established for each asphalt mixture to control experimental stochastic variability. The results were statistically aggregated, yielding the comparative performance parameters summarized in Table 7.

Analysis of Table 8 demonstrates the RA05 mixture exhibits exceptional high-temperature performance, achieving a dynamic stability of 22,318 cycles/mm at 70 °C, which is 64–218% higher than that of conventional materials. It is worth noting that at 70 °C, the stability of the RA05 mixture exceeds the 60 °C performance levels of other materials, with SMA-13 reaching 13,608 cycles/mm, CAM achieving 7880 cycles/mm, and AC-10 reaching 6986 cycles/mm.

Thermal sensitivity tests reveal progressive stability reductions (28.3–62.1%) across all materials when the temperature increases from 60 °C to 70 °C, emphasizing the distinct performance requirements for bridge deck pavements compared to standard road surfaces.

#### 3.2.2. Comparison of Different Structure Combinations

After evaluating individual materials, composite structures (SMA-13+RA05) underwent mechanical response testing using UTM-25. Comparative analysis with SMA-13+CAM and SMA-13+AC-10 configurations was performed cyclic loading (0.1 s half-sine waveform + 0.9 s rest interval) under 70 °C/0.7 MPa conditions. The termination criteria included 7000 loading cycles, an accumulated strain of 60,000 με, or the initiation of tertiary creep [39].

**Figure 9:**
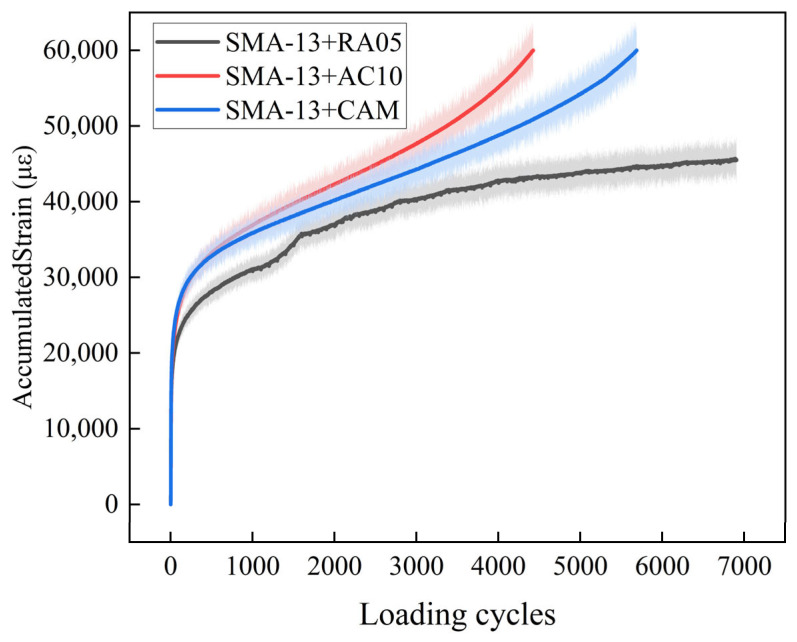
High-temperature dynamic creep test results.

Each structural configuration underwent three parallel tests (*n* = 3) to mitigate stochastic variability. The cumulative permanent strain curves were subsequently derived through the multivariate statistical integration of the resultant datasets. Figure 9 presents the cumulative permanent strain curves obtained from testing, with the corresponding regression equations and associated parameters for each deformation stage summarized in Table 8

As shown in Figure 9, under 70 °C testing conditions, both SMA-13+CAM and SMA-13+AC-10 configurations exceeded the critical strain threshold (60,000 με) within 4000 loading cycles. Comparatively, the SMA-13+RA05 composite maintained stable creep behavior through 7000 cycles with gradual strain progression.

As shown in Table 8, to investigate the influence of base course material type on the flow number of composite structures, an Analysis of Variance was conducted at a significance level of α = 0.05. The calculated *p*-value is significantly lower than 0.05 (*p* < 1.17 × 10^−5^), indicating demonstrated statistical significance that the high-temperature stability performance exhibits significant correlations with the type of base course material used. The value of $N_{ps}$s of the SMA-13 + RA05 composite structure is larger, which indicates that this composite structure enters the second stage later than the other two structures, while SMA-13+CAM (FN = 4700) and SMA-13+AC-10 (FN = 3600) both entered the third stage. Remarkably, the SMA-13+RA05 system achieved over 7000 cycles without entering the third stage, exhibiting 2.1–2.9 times greater rutting resistance than conventional counterparts.

This performance enhancement originates from RA05’s superior high-temperature stability in the ERS structural system, where its optimized viscoelastic properties effectively mitigate permanent deformation accumulation.

### 3.3. Temperature Reduction Measures for Bridge Deck Pavement Layers

Laboratory tests and thermal field simulations demonstrate stricter high-temperature requirements for steel bridge deck pavement materials compared to conventional road asphalt concrete. While standard specifications employ 60 °C for rutting tests, our analysis recommends elevating the testing threshold to 70 °C for bridge applications.

Lu et al. [40] identified significant thermal retention effects in enclosed steel box girders, where restricted air convection intensifies pavement layer heating.

Elevated temperatures induce significant performance degradation in the resin-dominated layer of ERS bridge deck pavement systems. Therefore, this study examines thermal regulation through controlled internal airflow modulation (0–2.4 m/s) within steel girders, maintaining structural integrity. Using finite element analysis software, thermal fields are recalculated under five airflow velocities, benchmarked against ambient wind conditions (2.4 m/s), seeking pavement temperature reduction methods without compromising girder stiffness or strength.

**Figure 10:**
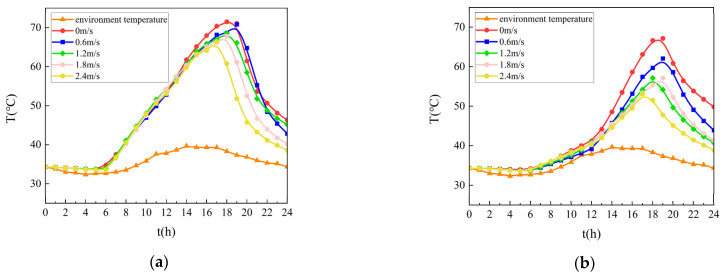
Temperature field distribution on the pavement in a day. (**a**) Temperature field distribution on the upper surface layer; (**b**) Temperature field distribution on the bottom surface layer.

The recalculated thermal distributions from 00:00 to 24:00 on a typical July day under varied ventilation scenarios are presented in Figure 10.

As illustrated in Figure 10, optimizing the ventilation within the steel box girder effectively reduces pavement surface temperatures, shifting the peak temperature time and shortening the duration of high-temperature exposure. Increased airflow velocity (0 → 2.4 m/s) reduced pavement surface peak temperatures by 8.3 while advancing peak occurrence time by 75 min, attributed to enhanced convective heat dissipation.

The thermal regulation effect became particularly pronounced during the 17:00–19:00 post-peak period. Open-girder configurations decreased high-temperature exposure (>50 °C) by 3 h (upper surface, Figure 10a) and 7 h (bottom surface, Figure 10b) compared to sealed conditions.

**Figure 11:**
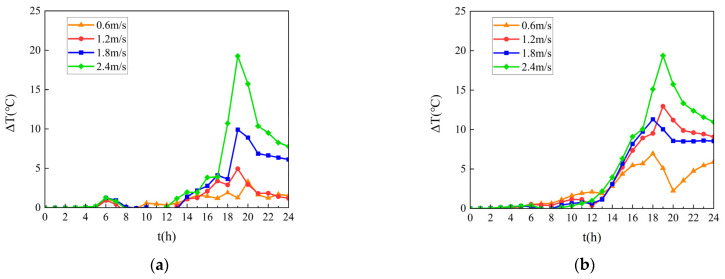
Temperature gradient evolution at the pavement in a day. (**a**) Temperature gradient evolution at the upper surface layer; (**b**) Temperature gradient evolution at the upper surface layer.

Figure 11 quantifies temperature reduction gradients from 00:00 to 24:00 on a typical July day through temporal mapping, revealing maximum cooling intensity of 14.2 °C at the pavement–girder interface under optimal ventilation conditions.

Figure 11 demonstrates that synchronous internal–external airflow velocities (open steel box girder configuration) achieve maximum cooling effectiveness with a 20 °C temperature reduction. A distinct temperature rebound phenomenon occurs at 09:00 under 0.6 m/s airflow conditions, where ventilation temporarily elevates pavement surface temperatures.

A comparative analysis of Figure 11a,b reveals more stable thermal regulation at the pavement base, maintaining continuous cooling from 10:00 without temperature inversion. This differential behavior originates from the thermal resistance characteristics of pavement materials, which amplify heat transfer constraints in lower structural layers.

Figure 11 reveals three distinct thermal regulation phases under ventilation control:

- **Initial Phase** (00:00–06:00). In the initial phase, the airflow impact is negligible due to the absence of solar loading, with the bridge system maintaining thermal equilibrium.
- **Diurnal Heating Phase** (06:00–19:00). In the diurnal heating phase, ventilation effectiveness escalates proportionally with solar intensity, achieving peak cooling of 20 °C in open configurations. Enhanced airflow (0.6–2.4 m/s) counteracts heat accumulation in enclosed girders through convective dissipation.
- **Nocturnal Cooling Phase** (19:00–24:00). In the nocturnal cooling phase, there is a progressive reduction in cooling rate (0.8 → 0.2 °C/h) as the structural thermal mass dominates heat transfer processes, independent of ventilation conditions.

This phased behavior highlights the time-dependent nature of thermal management in composite bridge systems, with solar loading intensity determining ventilation effectiveness thresholds.

## 4. Conclusions

This study investigates the high-temperature performance of ERS steel bridge deck pavement materials and calculates temperature fields through numerical simulations, yielding three principal conclusions:

(1) Under summer high-temperature conditions, the bridge deck pavement layer experiences significantly higher temperatures than the ambient air, with the peak surface temperature reaching up to 70 °C. This places greater demands on the high-temperature stability of pavement materials. The peak temperature at the bottom surface can reach 65 °C, necessitating strict assurance of bonding strength and shear resistance under elevated temperature conditions. Accordingly, it is recommended to raise the testing temperature for evaluating the high-temperature performance of pavement materials in steel bridge deck systems to approximately 70 °C.

(2) Laboratory results confirm the excellent high-temperature resistance of the ERS pavement system. The RA05 mixture achieved a dynamic stability of 22,318 cycles/mm at 70 °C, representing a 64–218% improvement over conventional materials. The SMA-13+RA05 composite structure sustained more than 7000 loading cycles, with rutting resistance more than twice that of traditional systems, primarily due to the superior properties of the RA05 layer.

(3) Enhancing internal airflow within the steel box girder effectively reduces peak pavement temperatures and high-temperature exposure durations. When the internal airflow matches ambient wind speed (open-girder configuration), surface temperatures can be lowered by up to 20 °C, and high-temperature durations can be shortened by 3–7 h. Implementing ventilation openings is thus an effective strategy to mitigate thermal stress without compromising girder stiffness or strength.

## Figures and Tables

**Table 1:** Technical parameters of SBS-modified asphalt [24].

Properties	Request	Results	Methods
Penetration (25 °C, 100 g, 5 s, 0.1 mm)	30~60	56	T0604-2011
Ductility (5 °C, 5 cm/min, cm)	≥30	43	T0605-2011
Softening point (TR&B, °C)	≥90	91.5	T0606-2011
After TFOT			
Mass loss (%)	≤1.0	0.193	T0610-2011
Penetration ratio (25 °C, %)	≥65	76.4	T0604-2011
Ductility (15 °C, cm)	≥10	21	T0605-2011

**Table 2:** Parameters of SMA-13.

Asphalt-Aggregate Ratio (%)	Theoretical Maximum Density (γt)/(g/cm^3^)	Bulk Density (γf)/(g/cm^3^)	Voids Volume (%)	Voids Volume (%)	Voids Filled with Asphalt (%)
6.0	2.584	2.476	4.0	16.1	75.4

**Table 3:** Technical parameters of RA binder [25].

Properties	Request	Results	Methods
Tack-Free Time (25 °C, h)	≥8.0	10.7	JT/T1131-2017 appendix C
Curing Time (25 °C, h)	≤72	53.0	JT/T1131-2017 appendix D
Elongation at Break (25 °C, %)	≥50	74	JT/T1131-2017 appendix E
Elongation at Break (−10 °C, %)	≥20	38	
Fracture Strength (25 °C, MPa)	≥2.0	2.4	JT/T1131-2017 appendix E
Fracture Strength (−10 °C, MPa)	≥5	5.7	

**Table 4:** Parameters of RA05.

Asphalt–Aggregate Ratio (%)	Bulk Density (γf)/(g/cm^3^)	Void Volume (%)	Marshall Stability (kN)	Flow Value (mm)
8.0	2.424	1.2	62.27	25.0

**Table 5:** Parameters of AC-10 and CAM.

Materials	Asphalt–Aggregate Ratio (%)	Theoretical Maximum Density (γt)/(g/cm^3^)	Bulk Density (γf)/(g/cm^3^)	Void Volume (%)	Void Volume (%)	Voids Filled with Asphalt (%)
AC-10	6.6	2.582	2.515	2.5	16.5	83.4
CAM	6.9	2.555	2.479	3.0	16.6	81.4

**Table 6:** Key simulation parameters of the computational model.

Item	Pavement Layers	Steel	Air
Thermal conductivity (J∙m^−1^∙h^−1^∙ °C^−1^)	4680	209,500	90
Density (kg/m^3^)	2300	7850	1.225
Specific heat (J/kg∙ °C)	925	460	1.005
Emissivity coefficient	0.90	0.55	-
Stefan–Boltzmann constant (J/h∙m^2^∙K^4^)	2.041092 × 10^−4^		
Interfacial thermal resistance (k∙m^2^/W)	0.00222		

**Table 7:** Rutting test results.

Temperature	DS (Cycles/mm)			
	SMA-13	RA05	AC-10	CAM
60 °C	10,624	30,014	7238	9347
70 °C	7697	22,318	6643	8567

**Table 8:** High-temperature dynamic creep test results of composite specimens.

Composite Specimens	Phase I	Phase II	$N_{ps}$	FN
SMA-13+RA05	$y=5283x^{0.2355}$	y=0.9595x+38,387	4250	>7000
SMA-13+CAM	$y=13,555x^{0.1589}$	y=5.6734x+34,609	1200	4700
SMA-13+AC-10	$y=15,591x^{0.1348}$	y=4.5058x+34,780	1200	3600

## Data Availability

The original contributions presented in this study are included in the article. Further inquiries can be directed to the corresponding author.

## Abbreviations

The following abbreviations are used in this manuscript:

SMA	Stone Mastic Asphalt
ERS	Epoxy Resin System
RA	Resin Asphalt
EBCL	Epoxy-Bonding Chip Layer
CAM	Crack-Attenuating Mixes
SBS	Styrene–Butadiene–Styrene
DS	Dynamic Stability
FN	Flow Number
